# Selenium Intake and Glycemic Control in Young Adults With Normal-Weight Obesity Syndrome

**DOI:** 10.3389/fnut.2021.696325

**Published:** 2021-08-19

**Authors:** Acsa de Castro Santos, Anna Flavia Ferreira Passos, Luciana Carla Holzbach, Cristiane Cominetti

**Affiliations:** ^1^Nutritional Genomics Research Group. Graduate Program in Nutrition and Health, School of Nutrition, Federal University of Goias, Goiania, Brazil; ^2^Nutrition Undergraduate Course, Federal University of Tocantins, Palmas, Brazil

**Keywords:** adults, adiposity, lifestyle, food consumption, glycated hemoglobin A

## Abstract

Numerous endogenous functions related to antioxidant processes, reproduction, and thyroid metabolism, as well as actions related to glycemic control, have been attributed to selenium. This study aimed to evaluate whether dietary selenium consumption is associated with variables of glycemic control in a sample of young Brazilian adults with Normal-Weight Obesity (NWO) syndrome. This was a cross-sectional study that evaluated 270 individuals with adequate body weight and excess body fat, who had their body composition assessed by dual-energy X-ray absorptiometry. Socioeconomic, health, and lifestyle questionnaires and three 24-h food records were applied. Glycemic control markers were also evaluated. The prevalence of inadequate selenium intake was analyzed by the Estimated Average Requirement (EAR) cut-point method. The prevalence of disturbances in glycemic control markers according to selenium consumption was compared by either the chi-square or the Fisher's exact test, with individuals classified according to the EAR values for selenium. The associations were evaluated by multiple linear regressions, using the backward strategy. The mean ± standard deviation (SD) age was 23.7 ± 3.3 years, and the mean ± SD daily selenium intake was 59.2 ± 26.4 μg. The overall prevalence of inadequate selenium intake was 59.2%. Individuals with selenium intakes below the EAR (≤45 μg/day) showed higher concentrations of glycated hemoglobin (HbA_1c_) (*P* = 0.002) and a higher prevalence of disturbances in HbA_1c_ than those with selenium intakes above the EAR (>45 μg/day) (*P* = 0.001). Dietary selenium intake was directly associated with female sex (β = 19.95, 95% CI 5.00 to 34.89; *P* = 0.001) and weight (β = 6.69, 95% CI 0.56 to 12.81; *P* = 0.010), and inversely associated with the percentage of total body fat (β = −0.80, 95% CI −1.56 to −0,04; *P* = 0.010) and HbA_1c_ (β = −7.41, 95% CI −13.06 to −1.75; *P* = 0.010). Considering the noticeable young age of the individuals evaluated and the high frequency of disturbances in HbA_1c_ concentrations in those with selenium consumption below the recommendation, it is suggested that adequate dietary intake or supplementation of this micronutrient should be guaranteed to prevent future possible complications associated with glycemic control disturbances.

## Introduction

Selenium is a trace element that fulfills key roles related to human body homeostasis, thyroid gland function, and optimal functioning of the immune system (1, 2). Most of these actions are due to its participation as a component or cofactor of antioxidant enzymes, such as glutathione peroxidase and thioredoxin reductase, in addition to deiodinases (1). Inadequate serum selenium concentrations are associated with disorders of the thyroid gland (2), cancer (3), metabolic syndrome, cardiovascular disease, diabetes (4, 5), and obesity—albeit in a contradictory way (6–8).

Obesity in the classic sense, defined by the Body Mass Index (BMI), has been extensively studied in several aspects, including selenium metabolism. However, more recently, emphasis has been placed on the substantial role of excess body fat regardless of BMI classification. In 2006, the Normal-Weight Obesity (NWO) syndrome, a metabolic condition of excess body fat in individuals with normal BMI, was defined (9). It is known that individuals with NWO have a particular profile concerning the development of some harmful conditions, with emphasis on cardiometabolic risk factors, such as insulin resistance (IR) and dyslipidemias. These markers of cardiometabolic health are among the important aspects that should be evaluated and monitored in individuals with NWO because this condition seems to favor metabolic disorders in an intermediary way between individuals with normal BMI and body composition and those with obesity. Therefore, the role of dietary selenium intake in glycemic control in these individuals should also be investigated.

Although the most selenium-rich food is widely available in Brazil, there are few studies on the consumption of selenium in the Brazilian population and none in individuals with NWO. Analysis of the dietary intake of 34,003 Brazilians older than 10 years revealed a mean selenium intake of 107.6 μg/day, with no difference between income classes or urban and rural areas. Higher selenium intakes were found in women and residents of the northern region of Brazil. Among the elderly, mean selenium intake was lower than that in other age groups but still above the RDA (10), in contrast to the results of a study on elderly residents in the Rio Grande do Sul state, southern Brazil, which found an inadequate intake rate of 98% (11).

In general, studies investigating the relationship between selenium and glycemic control show contradictory results regarding glycemia and the prevalence of type 2 diabetes mellitus (DM2) but converge concerning serum insulin and the Homeostasis Model Assessment-Insulin Resistance (HOMA-IR) index and suggest that its action occurs through some mechanisms, including regulation of insulin signaling and glycolysis, pyruvate, and chromium metabolism (7, 12–15), as well as promoting changes in the expression of genes related to insulin and adiponectin receptors (*INSR* and *ADIPOR1*) and others that encode pyruvate metabolism enzymes (*LDH, PDHA, PDHB*) (16, 17).

In addition, it is believed that selenium could play a protective role against disorders of glucose metabolism by regulating oxidative stress and mimicking insulin action (18, 19). However, important results of a study with data from samples of American individuals (14) showed that selenium is associated with a higher risk of DM2. Therefore, it is currently believed that high serum selenium concentrations increase the risk of insulin resistance or DM2 (20). One explanation is that selenoprotein glutathione peroxidase 1 (GPX-1) is overexpressed under high selenium concentrations (21). Overexpression of GPX-I has been associated with insulin resistance in rats (22) and decreased expression of *PKM*, the gene encoding the glycolytic enzyme pyruvate kinase (23).

In a recent meta-analysis with studies including individuals from Europe and Asia with diseases such as DM2, gestational diabetes, polycystic ovary syndrome, heart disease, obesity, and others, selenium supplementation was shown to decrease the Homeostasis Model Assessment of Beta-Cell Function (HOMA-beta) and increase the Quantitative Insulin Sensitivity Check Index (QUICKI); however, it had no effect on blood glucose, HOMA-IR, glycated hemoglobin (HbA_1c_), and adiponectin concentrations (8). In addition, after 16 years of monitoring more than 10,000 women in Italy, it was observed that those who developed diabetes, among other factors, had higher dietary selenium intake (60.9 vs. 56.8 μg/day) (24).

Considering that the association of dietary selenium intake with markers of glycemic control has produced variable results depending on the study population and that a similar investigation has not yet been carried out in adults with NWO, this study aimed to evaluate whether dietary selenium consumption is associated with glycemic control variables in a sample of young adults with NWO. The hypothesis is that selenium consumption is associated with serum concentrations of glucose; insulin; HbA_1c_; and the HOMA-IR, HOMA-beta, and QUICKI indexes in these individuals.

## Materials and Methods

### Design and Population

**Figure 1:**
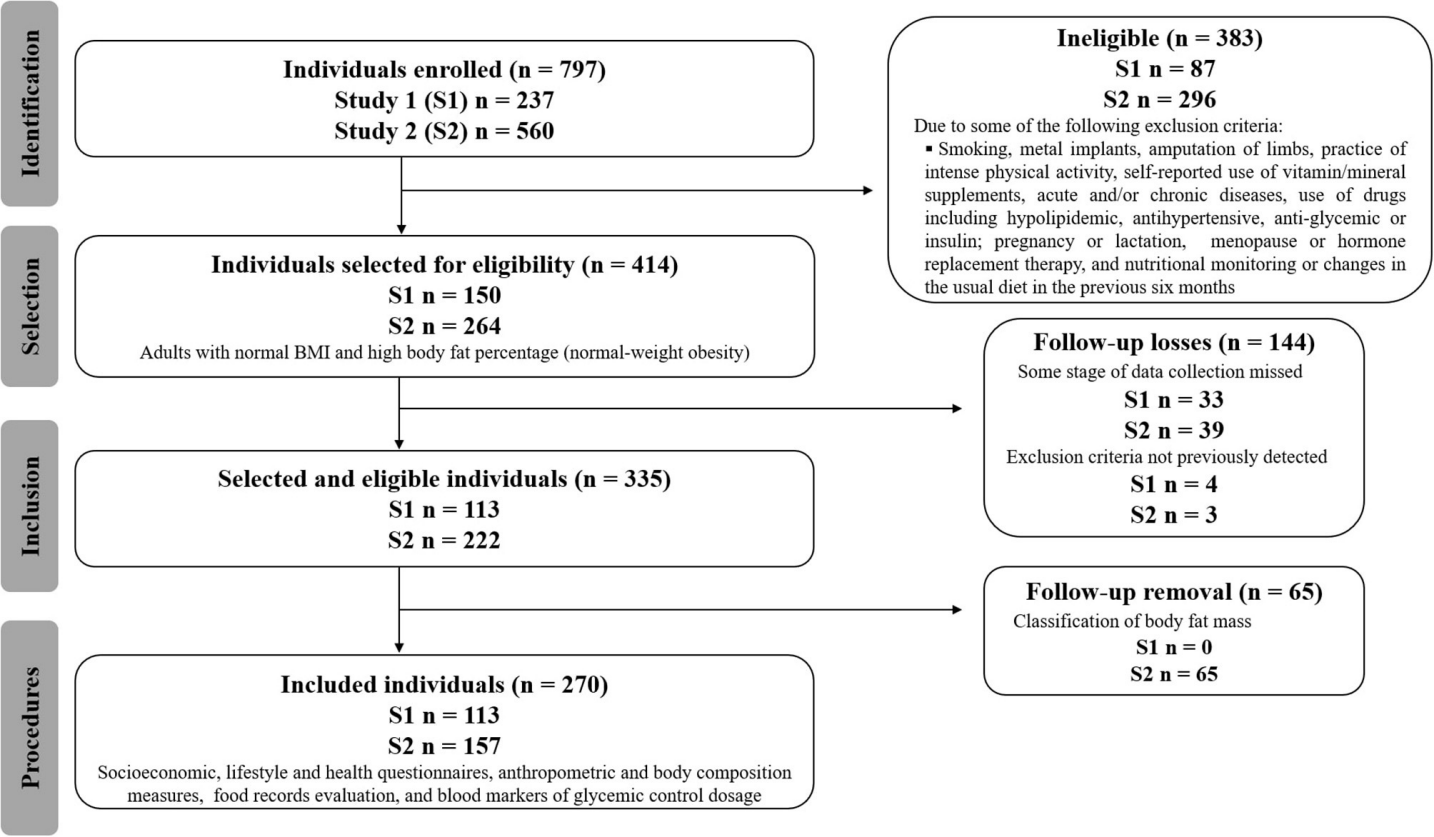
The Strengthening the Reporting of Observational Studies in Epidemiology (STROBE) flow-chart of participants.

This study was carried out using two databases, applying the same inclusion and exclusion criteria. Both databases came from observational, analytical, and cross-sectional studies, collected from an academic community in Goiânia, Goiás, Brazil. The recruitment of volunteers was carried out through folders, social networks, and emails sent to students, employees, and professors at the Federal University of Goias (UFG), Brazil (Figure 1). The studies were approved by the UFG Research Ethics Committees (protocols n°. 2,772,022, Jul 16, 2018 and n° 865.062, Nov 10, 2014). All volunteers received information and clarification about all procedures and signed a consent form.

Both men and women aged between 20 and 59 years, with normal BMI (between 18.50 and 24.99 kg/m^2^) (25) and high body fat percentages (9, 25) were included. One hundred thirteen individuals were recruited in the first study and 157 in the second study, totaling 270 volunteers (Figure 1). The statistical power was estimated using R software, with an effect size of 0.21, a type I error probability of 0.05, and a sample size of 270 observations. The estimated power was 0.858.

### Study Population and Measurements

The first data collection was performed from May to September 2015, and the second one, from January to June 2019. Data collection took place in two meetings after participant recruitment. During the first appointment, individuals received all information about the research, and the informed consent form was presented. Individuals who met the inclusion criteria and agreed to participate answered the socioeconomic, demographic, lifestyle, and health questionnaires and were referred for anthropometric assessment, body composition, and food consumption analysis. The second appointment was previously scheduled, and the individuals were instructed to fast for 12 h to collect blood samples for biochemical tests.

All researchers on the team in both studies were trained and underwent standardization regarding the nutritional care protocol. In addition to this training, the researchers received training to measure anthropometric measures, according to the technique of anthropometry standardization recommended by Habicht (26).

### Socioeconomic, Demographic, Lifestyle, and Blood Pressure Data

The application of a questionnaire referring to socioeconomic, demographic, and lifestyle data took place in an average of 30–40 min. During this stage, blood pressure was measured at three non-consecutive moments, and the mean values of systolic and diastolic pressures were used in the analyses (27).

### Anthropometric Evaluation

Body mass was measured on a digital platform scale (Filizola Shop, São Paulo, Brazil), with a maximum load of 150 kg and an accuracy of 0.1 kg. Height was determined using a stadiometer (Seca Deutschland, Hamburg, Germany) with a maximum reach of 220 cm and a precision of 0.1 cm, according to the procedures described by Lohman et al. (28).

### Body Composition Analysis

Individuals dressed in light clothing and without accessories were subjected to DEXA examination, using a Lunar DPX NT model DEXA scanner (General Eletric Medical Systems Lunar® Madison, USA).

### Determination of Glycemic Profile Markers

Blood was collected by a specialized technician, with disposable materials, in a strictly sanitized environment, from the median cubital vein for determination of the glycemic profile (fasting blood glucose, fasting insulin, and HbA_1c_).

The colorimetric enzymatic method was used to determine the fasting blood glucose values, and the reference value for disturbances was ≥100 mg/dL (29). The electrochemiluminescence method was applied to determine the serum insulin concentration.

The immunoturbidimetric inhibition method was used to determine the concentration of HbA_1c_, and the percentage was calculated using the equation [(28.7 × HbA_1c_) −46.7]. Results ≥5.7% were considered altered (29).

HOMA-IR was calculated according to the equation proposed by Matthews et al. (30): HOMA-IR = (FPI × FPG)/22.5, in which FPI refers to fasting plasma insulin and FPG, to fasting plasma glucose. This index assesses IR and was considered above normal when higher than 2.71 (29). The HOMA-beta index estimates the functional capacity of pancreatic beta cells and was calculated according to the equation proposed by Matthews et al. (30): HOMA-beta = (20 × FPI)/(FPG −3.5). Values above the 90th percentile of the sample were considered above normal. For the sample in this study, the cutoff point was >223.56.

The Quantitative Insulin sensitivity Check Index (QUICKI) was calculated from results of insulinemia and fasting glycemia using the equation: [1/(log FPI + log FPG)]. This index assesses insulin sensitivity, and the value adopted as a reference for altered values was less than the 10th percentile of the sample (29). For this study, the cutoff point was 0.3305.

### Food Intake Analysis

Dietary food intake was assessed using three 24-h food records (R24H), on alternate days and different weeks, including a weekend day (31). Aiming at reducing possible collection errors, R24H were applied following the Multiple Pass Method (MPM) (32), which helps the interviewee to remember in detail the food and drinks consumed the day before the collection. In addition, strategies were used to assist in the measurement of portions, such as a photographic manual and standard tools for home measurements (33). The first R24H was applied at the first appointment, and the research team contacted the participants to collect the other R24h. These assessment instruments were applied by two nutritionists who underwent technical and practical training for the collection and evaluation of R24h.

The information collected was transformed into standard home measures, and the data evaluation was carried out with a Brazilian software (Avanutri®, Três Rios, Rio de Janeiro, Brazil). This software calculates the results of 21 micronutrients, including selenium, in micrograms. Selenium intake was adjusted to the energy value, according to the residual method (34). In summary, this method results in the estimation of the residual value of a regression model in which the independent variable is the total energy intake and the dependent variable is the raw consumption of the nutrient under analysis. Therefore, the residual value reflects an estimate of nutrient intake not correlated with total energy intake and directly related to general variation in food choice and composition.

### Statistical Analysis

A double-entry database was developed to check the consistency of the results. Descriptive analysis, including mean ± SD or median (interquartile range – IQR), was performed for all quantitative variables. Shapiro–Wilk's W test was applied to evaluate the significance (α = 0.05) of normality deviations observed in the residuals of mean comparisons tests and linear regression models.

Student's *t*-test or the Mann–Whitney test was applied to compare means. A comparison of the disturbances in glycemic control markers between individuals consuming selenium at levels below and above the EAR was performed using either the chi-square or Fisher's exact test, according to the number of individuals who presented disturbances. Associations between variables were assessed by multiple linear regression models, using the backward strategy. The variables that could associate with glycemic response and were added to the model included sex, weight, height, BMI, body fat percentage, and HbA_1c_, age, systolic and diastolic blood pressure, waist circumference, android and gynoid fat, the android/gynoid ratio, fasting blood glucose, insulinemia, and the HOMA-IR, HOMA-beta, and QUICKI indexes. Although selenium intake was the main focus of the analysis, other dietary variables that also could interfere with glycemic response were added to the model, including carbohydrate, total fat; saturated, monounsaturated and polyunsaturated fatty acids; and protein intakes.

A value of *P* < 0.05 was considered statistically significant, and all analyses were performed in R software version 4.0.3 (35).

## Results

The total sample consisted of 270 adults (113 individuals from the study conducted in 2015 and 157 from the one conducted in 2019) (Figure 1), with NWO (adequate BMI and a high percentage of body fat). The mean ± SD age was 23.7 ± 3.3 years, and 70.4% of the participants were women.

For selenium, individuals showed a mean intake of 59.2 ± 26.4 μg/day. The overall prevalence of inadequate intake was 59.2% (54.7% for men and 63.7% for women). To analyze glycemic control marker disturbances, individuals were separated into two groups according to selenium consumption: (1) below the EAR (≤45 μg/day)—91 individuals (33.7%); and (2) above the EAR (>45 μg/day)—179 individuals (66.3%) (Table 1). When comparing the total sample according to sex, 30.5% (*n* = 58) of women and 41.2% (*n* = 33) of men showed selenium consumption below the EAR (*P* = 0.089).

**Table 1:** Descriptive data of individuals with Normal-Weight Obesity Syndrome, according to the classification of dietary selenium intake.

	**Below EAR (*n* = 91–33.7%)**	**Above EAR (*n* = 179–66.3%)**	**Total (*n* = 270–100.0%)**	***P*-value**
Sex				0.089
Male	33 (36.3)	47 (26.3)	80 (29.6)	
Female	58 (63.7)	132 (73.7)	190 (70.4)	
Age (years)	22.4 (21.1–26.3)	23.0 (21.2–25.0)	23.0 (21.2–25.0)	0.879
Selenium intake	33.4 ± 9.3	72.4 ± 22.2	59.2 ± 26.4	0.001
MSBP (mmHg)	108.4 ± 11.3	107.0 ± 10.8	107.5 ± 11.0	0.343
MDBP (mmHg)	65.5 (60.5–72.0)	65.0 (60.0–72.0)	65.0 (60.0–72.0)	0.606
Weight (kg)	61.2 (56.5–66.8)	60.6 (55.2–67.0)	61.0 (55.4–66.8)	0.649
Height (m)	1.7 (1.6–1.8)	1.7 (1.6–1.7)	1.7 (1.6–1.7)	0.481
BMI (kg/m^2^)	22.5 ± 1.6	21.8 (20.7–23.2)	22.0 (21.0–23.4)	0.476
MET (min/week)	400.0 (120.0–898.0)	297.0 (140.0–565.0)	330.0 (132.8–726.0)	0.062
%BF	34.7 (26.5–40.0)	35.2 (30.4–38.8)	35.0 (29.4–39.2)	0.672
%AF	37.5 (31.6–43.1)	35.2 (30.2–41.8)	35.8 (30.8–42.4)	0.308
%GF	46.2 (34.4–50.7)	47.1 (41.0–50.3)	46.7 (38.3–50.4)	0.475
A/G	0.9 ± 0.1	0.8 ± 0.1	0.8 ± 0.1	0.005

*Data are absolute numbers (percentages), mean ± standard deviation or median (interquartile range). MSBP, mean systolic blood pressure (mmHg); MDBP, mean diastolic blood pressure (mmHg); BMI, body mass index; MET, metabolic equivalent of task; %BF, body fat percentage; %AF, android body fat percentage; %GF, gynoid body fat percentage; A/G, ratio between %AF/%GF. Student's t-test or Mann-Whitney test*.

There were no differences in weight, BMI, waist circumference, body fat percentage (% BF), android fat, and gynoid fat between the two groups (below and above the EAR). Individuals in the group below the EAR showed higher values of the ratio between android/gynoid body fat compared to those in the group above the EAR (Table 1).

Individuals in the group with selenium intake above the EAR had lower concentrations of HbA_1c_ than those in the group with intake below the EAR (*P* = 0.002) (Table 2). In addition, a higher prevalence of disturbances in HbA_1c_ concentrations was found in the group with selenium consumption below the EAR compared with the one with intake above EAR (*P* = 0.001) (Table 3). No differences were found in the other biomarkers of glycemic control between the two groups (Table 3).

**Table 2:** Biochemical characterization of individuals with Normal-Weight Obesity Syndrome, according to dietary selenium intake.

	**Below EAR (*n* = 91–33.7%)**	**Above EAR (*n* = 179–66.3%)**	**Total (*n* = 270–100.0%)**	***P*-value**
Blood glucose (mg/dL)	85.5 ± 7.3	85.2 ± 7.5	85.3 ± 7.4	0.800
Insulin (uU/mL)	7.2 (5.4–9.9)	7.2 (5.0–9.4)	7.2 (5.1–9.8)	0.443
HOMA-IR	1.6 (1.1–2.2)	1.5 (1.0–2.1)	1.5 (1.1–2.1)	0.384
HOMA-Beta	122.9 (83.8–164.5)	118.3 (88.3–171.6)	122.0 (86.5–169.0)	0.881
HbA_1c_%	5.1 (4.7–5.6)	4.8 (4.6–5.1)	4.9 (4.6 −5.3)	0.002
QUICKI index	0.36 (0.34–0.38)	0.36 (0.34–0.38)	0.36 (0.34–0.38)	0.387

*Data are mean ± standard deviation or median (interquartile range). HOMA-IR, homeostasis model assessment of insulin resistance; HOMA Beta, homeostatic model assessment of beta-cell function; HbA_1C_, glycated hemoglobin. Student's t-test or Mann-Whitney test*.

**Table 3:** Disturbances in biomarkers of glycemic control of individuals with Normal-Weight Obesity Syndrome, according to dietary selenium intake.

	**Below EAR (*n* = 91)**	**Above EAR (*n* = 179)**	**Total (*n* = 270)**	***P*-value**
Blood glucose	2 (2.2)	9 (5.0)	11 (4.1)	0.344
HOMA-IR	6 (6.6)	18 (10.1)	24 (8.9)	0.345
HOMA-beta	7 (7.7)	20 (11.2)	27 (10.0)	0.366
HbA_1c_	20 (22.0)	15 (8.4)	35 (11.5)	0.001
Quicki index	8 (8.8)	19 (10.6)	27 (10.0)	0.637

*Data are absolute numbers (percentage). HOMA-IR, homeostasis model assessment of insulin resistance; HOMA-beta, homeostatic model assessment of beta-cell function; HbA_1c_, glycated hemoglobin. Fisher's exact test or Chi-square test*.

Three individuals were excluded from the regression analysis due to missing data (*n* = 267). After all candidate variables were added into the multiple regression model using the backward strategy, the final model with the lowest Akaike criterion presented the variables sex, weight, height, BMI, body fat percentage, and HbA_1c_. Sex, weight, body fat percentage, and HbA_1c_ were the variables most strongly associated with dietary selenium intake. The female sex was directly associated with a higher consumption of selenium (β = 19.95, 95% CI 5.00 to 34.89; *P* = 0.001), and a positive association was also observed for selenium consumption and weight (β = 6.69, 95% CI 0.56 to 12.81; *P* = 0.010). However, negative associations of body fat percentage and HbA_1c_ with selenium consumption were observed (β = −0.80, 95% CI −1.56 to −0,04; *P* = 0.010, and β = −7.41, 95% CI −13.06 to −1.75; *P* = 0.010, respectively). The final regression model is shown in Table 4. The variables that sequentially left the model during the backward procedure to derive the final regression model were age; systolic blood pressure; diastolic blood pressure; waist circumference; android fat; gynoid fat; the android/gynoid ratio; fasting blood glucose; insulinemia; and the HOMA-IR, HOMA-beta, and QUICKI indexes. It is important to highlight that the regression model was also tested only for females, considering the highest percentage of women in the sample; however, as the results were not different from those observed in the analysis of both sexes combined (data not shown), it was decided to present the results of the total sample. In addition, adding macronutrients, saturated, monounsaturated, and polyunsaturated fatty acids to the regression model did not change the association between dietary selenium intake and HbA_1c_.

**Table 4:** Associations among selenium intake and explanatory variables, determined by multiple regression model adjusted by the backward strategy (*n* = 267).

	**Coefficient (β)**	**SE**	**95% CI**	***t* value**	**pr (>|t|)**	***P* value**
Intercept	785.2937	389.01	19.28–1551.31	2.019	0.044^*^	0.01
**Variables**						
Female sex	19.9459	7.59	5.00 to 34.89	2.628	0.009^**^	0.001
Weight	6.6873	3.11	0.56 to 12.81	2.150	0.032^*^	0.010
Height	−450.6025	231.24	−905.95 to 4.75	−1.949	0.052^·^	0.050
BMI	−15.2345	8.77	−32.50 to 2.03	−1.737	0.084^·^	0.050
%BF	−0.8027	0.39	−1.56 to −0.04	−2.072	0.039^*^	0.010
HbA_1c_	−7.4051	2.87	−13.06 to −1.75	−2.579	0.010^*^	0.010

*Significance codes:*

* *0.01*,

** *0.001*,

*·0.05*.

*Residual standard error: 25.59 on 260 degrees of freedom (DF)*.

*Multiple R-squared: 0.08447*.

*Adjusted R-squared: 0.06334*.

*F-statistic: 3.998 on 6 and 260 DF*.

***P-value: 0.000757***.

*BMI, body mass index; %BF, body fat percentage; HbA_1c_, glycated hemoglobin*.

## Discussion

The assessment of risk markers for metabolic diseases in individuals with NWO is important to reduce the medium and long-term risks of cardiovascular diseases and DM2. In a quick search of the scientific literature, it is possible to identify many studies that investigate the relationship between selenium supplementation and glycemic control markers; however, few report this association for food consumption, and none has been carried out with individuals with NWO.

Our results show that mean dietary selenium consumption was above that recommended by the DRIs (59.2 ± 26.4 μg/day); however, approximately one third of participants did not reach the expected minimum values. We also observed that dietary selenium intake was inversely associated with serum concentrations of HbA_1c_ and body fat percentage. Individuals with intake above the EAR for selenium had lower concentrations of HbA_1c_, which may suggest better glycemic control. Although HbA_1c_ should not be used alone for DM2 diagnosis or glycemic profile monitoring, it is extremely relevant in clinical practice, as it allows for screening of mean blood glucose in the last 90 days (36). The association with body fat percentage draws attention to the need of monitoring this parameter in addition to the BMI.

Studies about the action of selenium on glycemic control have produced divergent results. A recent meta-analysis of randomized clinical trials (RCTs), including 1,441 individuals with ages ranging from 10 to 85 years, verified that selenium supplementation reduced insulin secretion as assessed by the HOMA-beta index and increased the QUICKI index; however, significant results were not observed for disturbances in HOMA-IR and HbA_1c_ concentrations (8). On the other hand, in a study with 2,420 non-diabetic Canadians (mean age of 42 years), dietary selenium intake was positively associated with glycemic control, so that insulin resistance, measured by HOMA-IR and HOMA-beta, decreased with increasing selenium consumption among women but not among men (*P* < 0.001); lower daily selenium consumption was observed among those with the highest HOMA-IR indexes (*P* < 0.001) and a negative correlation between selenium and insulin resistance was observed up to a consumption limit of 1.6 μg/kg/day (37). Although this study shows relevant data, the mean dietary consumption of selenium (109.22 ± 1.18 μg/day) was 1.84 times higher than that observed in our study, which makes comparisons of results difficult.

Two RCTs on selenium supplementation carried out in Danish elderly and Polish adults reinforce our main result that higher intakes of selenium are associated with lower concentrations of HbA_1c_, even within different populations and with different doses of selenium (16, 38). By contrast, some studies have failed to demonstrate a decrease in HbA_1c_ concentrations (12, 39) or found high serum selenium concentrations associated with increased concentrations of glucose and HbA_1c_, as in 41,474 American adults with a mean dietary selenium intake of 98.0 ± 55.0 μg/day (40) and in 8,824 Chinese with a mean consumption of 52.4 μg/day (41).

Some mechanisms to justify the controversial action of selenium in relation to the processes associated with glucose metabolism are speculated. An *in vivo* study with diabetic mice showed that the use of sodium selenate increased the expression of peroxisome proliferator-activated receptor gamma (PPAR-γ), which acts on insulin resistance and increases lipid metabolism (42). The selenium compounds of the oxidation state ^+^IV inhibited the activity of protein tyrosine phosphatases (PTP) (42), responsible for dephosphorylating the insulin receptor, attenuating its action (43). In this context, selenium as sodium selenite would antagonize and compromise glycemic control.

Robertson and Harmon (44) and Campbell et al. (45) suggest that selenium could provide protection against DM2 due to its association with oxidative stress control by increasing the activity of GPx in pancreatic beta cells and decreasing the damage caused by reactive oxygen species (ROS). This was verified in a mouse beta-cell line (Min6) in which beta-cell sensitivity increased in response to sodium selenate, expressed via increased GPx activity (5-fold), and a notable increase in the activity of insulin promoter factor 1 (*Ipf1*). A response to sodium selenite was also observed via increased mRNA levels of *Ipf1* and *Ins*. The increased antioxidant activity of GPx culminates in beneficial effects on the level of pancreatic beta-cell mass and insulin synthesis; however, long-term increased insulin production and secretion can result in chronic hyperinsulinemia (46).

In our study, in addition to the results observed in relation to HbA_1c_ concentrations, positive associations were found between dietary selenium consumption with female sex and weight, and an inverse association between selenium intake and body fat percentage was observed. The Canadian CODING study with 3,054 participants (adults from the province of Newfoundland, in eastern Canada, mean age of 42 years), observed a mean dietary selenium consumption of 108.10 μg/day, and decreasing consumption in μg/kg/day along the BMI ranges. The authors found a significant and dose-dependent association between tertiles of selenium consumption and weight, BMI, waist circumference, total body fat percentage, and gynoid and android fat percentages and demonstrated that dietary selenium can account, regardless of other factors, for 9–27% of the variation in body fat percentage (47).

Thereafter, a study published by the same group of CODING (*n* = 2,420), showed divergent results regarding the type of association between dietary selenium consumption and body weight but also observed an inverse association with body fat percentage, BMI, and body water (37). A similar result was also found in a study with a representative national sample of Chinese individuals, in which the body fat percentages were also lower in individuals consuming selenium in the highest quintiles compared with those with intake in the lowest quintiles (41).

Possible explanations for the role of selenium in adipogenesis are also related to PPAR-γ, which is responsible for adipocyte differentiation and distribution of adipose tissue, and is thus related to increased tissue insulin sensitivity and weight control. In animals receiving sodium selenate, hepatic PPAR-γ expression was 2.5 times higher than in those with selenium deficiency or treated with sodium selenite (42). On the other hand, a recent study with rat and human cell cultures of pre-adipocytes showed inhibition of adipogenesis due to a decrease in the expression of PPAR-γ and fatty acid synthase mRNAs, while there was an increase in growth factor-β (48). The mechanisms involved in the association of selenium with adiposity are not yet fully elucidated, but there is evidence suggesting that serum selenium concentrations are negatively associated with adiposity, especially visceral fat. However, this information needs to be carefully considered because it involves numerous other processes and not only dietary selenium intake as a modifier of serum concentration (49).

Among the limitations of our study, the cross-sectional design can be considered, as it does not allow us to make inferences about cause and effect between dietary selenium consumption and the other markers. However, it suggests that there may be interactions between the consumption of this nutrient and glycemic control markers and, for this reason, may guide future investigations on dietary consumption and the effect of selenium on biochemical and metabolic pathways and the expression of genes related to variation in HbA_1c_ concentrations. We were not able to measure blood selenium levels, which can also be considered a limitation. On the other hand, this is the first study to assess associations between dietary selenium intake and markers of glycemic control in individuals with NWO.

In conclusion, dietary consumption of selenium above the minimum values recommended for groups by the dietary guidelines was associated with lower serum HbA_1c_ concentrations, a long-term marker of disturbances in blood glucose, and body fat percentage. Considering that the individuals evaluated in our study had NWO, and the disturbances in HbA_1c_ found in the group consuming selenium below the EAR, we can observe that despite their noticeably young age, these individuals showed cardiometabolic markers that are more commonly seen in older people. Therefore, further studies with individuals with NWO are strongly recommended, as one of the basic principles of nutrition is to reduce or mitigate the risk of non-communicable chronic diseases throughout life.

## Data Availability Statement

The raw data supporting the conclusions of this article will be made available by the authors, upon a reasonable request.

## Ethics Statement

The studies involving human participants were reviewed and approved by UFG Research Ethics Committee. The patients/participants provided their written informed consent to participate in this study.

## Author Contributions

AS and AP: acquisition, interpretation, and analysis of data. LH: data interpretation and writing the manuscript. CC: conceptualization, experimental design, obtaining resources, interpretation of data, and writing the manuscript. All authors read and approved the final manuscript.

## Conflict of Interest

The authors declare that the research was conducted in the absence of any commercial or financial relationships that could be construed as a potential conflict of interest.

## Publisher's Note

All claims expressed in this article are solely those of the authors and do not necessarily represent those of their affiliated organizations, or those of the publisher, the editors and the reviewers. Any product that may be evaluated in this article, or claim that may be made by its manufacturer, is not guaranteed or endorsed by the publisher.
